# Association of Insufficient or Excess Sleep with Type 2 Diabetes Mellitus in the Presence of Periodontitis

**DOI:** 10.3390/ijerph17207670

**Published:** 2020-10-21

**Authors:** Se-Yeon Kim, Ji-Soo Kim, Min-Ji Byon, Hyun Kyung Kang, Jin-Bom Kim

**Affiliations:** 1 Department of Preventive and Community Dentistry, School of Dentistry, Pusan National University, 49 Busandaehak-ro, Yangsan 50612, Korea; secan00@naver.com (S.-Y.K.); psily1@naver.com (J.-S.K.); kyura2@naver.com (M.-J.B.); 2 BK21 FOUR Project, School of Dentistry, Pusan National University, Yangsan 50612, Korea; 3 Department of Dental Hygiene, Silla University, Baekyang-daero, 700, Sasang-gu, Busan 46958, Korea; kanghk75@gmail.com

**Keywords:** sleep, inadequate sleep, excessive sleep, impaired fasting glucose, type 2 diabetes mellitus, periodontitis

## Abstract

We aimed to investigate the effects of sleep duration on impaired fasting glucose and diabetes in Korean adults with periodontal disease. This cross-sectional study was performed using data for 10,465 subjects aged >19 years who completed the periodontal examination and questionnaires in the Sixth Korea National Health and Nutrition Examination Survey (2013–2015). The effect of sleep was confirmed by a complex-sample multinomial logistic regression analysis. Confounding variables were age, sex, household income, education level, smoking status, and sleep duration. Of all participants, 25.7% had periodontitis, of which 28.6% had fasting serum glucose disorder and 14.2% had diabetes. Among participants with periodontitis, the prevalence of diabetes was 1.49 times higher in participants with an average sleep duration of ≥8 h than those with an average sleep duration of 6–7 h. The prevalence of diabetes among participants without periodontitis was 1.49 times and 1.57 times higher in participants with an average sleep duration of ≤5 and ≥8 h, respectively, than those with an average sleep duration of 6–7 h. We found that altered sleep duration may be a risk factor for diabetes and that proper sleep duration is important to control diabetes incidence.

## 1. Introduction

Stress, increasing activity time, and advancements in electronic devices have resulted in major sleep deficits in modern society [1]. People sleep one-third of their lifetime. According to a simple behavioral definition, sleep is a reversible behavioral state of perceptual disengagement from and unresponsiveness to the environment [2]. The circadian sleep rhythm is considered to influence sleep habits [3]. An adequate amount of good-quality sleep affects immune system response and body functioning, so high-quality sleep duration is a very important parameter for health enhancement and maintenance [4]. Moreover, lack of sleep can result in physical and psychological disorders [5].

Inappropriate sleep duration and poor sleep quality have been recently reported to be risk factors for diabetes mellitus (DM) [6,7]. Too much or too little sleep disrupts glycemic control in both prediabetes and type 2 diabetic patients. In addition, inadequate sleep duration increases the risk of obesity and metabolic syndrome and can increase mortality rates [8].

Sleep duration has also been associated with the prevalence of dyslipidemia [9]. Sleep durations above or below the median of 7 to <8 h per night are associated with an increased prevalence of hypertension. Circadian misalignments such as shift work are also associated with an increased risk of developing type 2 diabetes [10]. Korean adults have an average sleep duration of 6.8 h, which is less than the sleep duration in OECD (Organization for Economic Cooperation and Development) countries [11].

Diabetes is a group of chronic metabolic diseases characterized by hyperglycemia resulting from defects in insulin secretion, insulin action, or both. Periodontal disease is known as the sixth most common complication of DM [12,13]. Diabetic patients have been shown to present with a higher prevalence and severity of periodontal disease than non-diabetic patients [14].

The most evident change in patients with diabetes is the decrease in external defense mechanisms and the resultant increased susceptibility to infections. The major signs in the oral cavity are changes in periodontal tissues; gingival enlargement; gingival polyps; formation of abscess; gingival bleeding; periodontal disease; tooth mobility [15]. The prevalence of periodontitis in patients whose serum glucose level was not regulated was higher than that in individuals with regulated glucose levels and patients without glycemic control showed increased severity of periodontal disease [16]. Regardless of the type of diabetes, diabetic patients experience a significant increase in periodontal tissue destruction. Mattout et al. reported that compared to non-diabetic patients, diabetic patients showed greater destruction of periodontal tissues such as gingivitis and clinical attachment loss (CAL) [17].

The risk of diabetes has also been shown to increase with chronic periodontal disease [18]. The pathophysiologic mechanism of the association between periodontal disease and diabetes is likely to be complex and bidirectional [19]. The purpose of this study was to investigate the effects of sleep duration on impaired fasting glucose and diabetes in participants with periodontal disease.

## 2. Materials and Methods

### 2.1. Data Sources

This study was based on data obtained from the Sixth Korea National Health and Nutrition Examination Survey (KNHANES-VI), which was conducted from 2013 to 2015 by the Korea Centers for Disease Control and Prevention. KNHANES is conducted annually using a rolling sampling design, which involves a complex, stratified, multistage probability-cluster survey of a large representative sample of the non-institutionalized civilian population in South Korea. The purpose of this survey was to gather national data about the health status and behaviors as well as the nutritional intake of the Korean population. KNHANES-VI included highly structured health-related questionnaires, a nutrition survey, and an oral health examination conducted by trained dentists.

**Figure 1:**
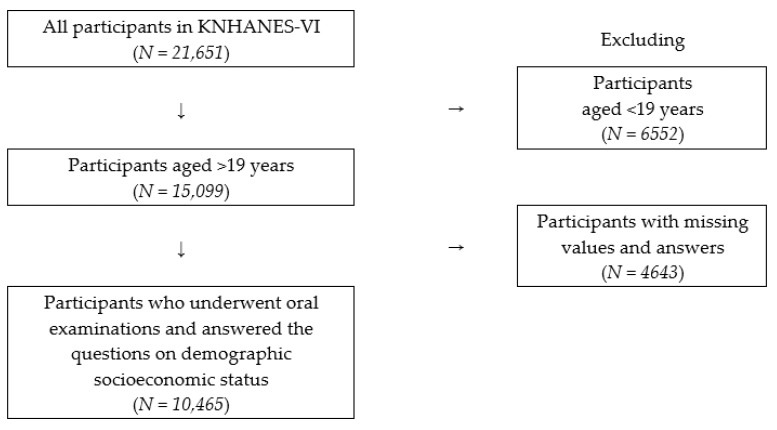
Flowchart for sampling of study participants (KNHANES-VI: Sixth Korea National Health and Nutrition Examination Survey).

This cross-sectional analysis was restricted to adult participants aged >19 years, among all 15,099 participants in KNHANES-VI, who had undergone the oral health examination and completed the health-related questionnaires (20,219,595 weighted samples). Participants with one or more missing answers in the questionnaires and participants without oral health examination data were excluded from the analysis. We eventually included 10,465 individuals who had undergone clinical oral health examinations and completed the questionnaires, which included data for demographic socioeconomic variables, sleep duration, diabetic status, and prevalence of periodontal disease from KNHANES-VI (Figure 1).

All participants gave their informed consent for inclusion before their participation in the study. The study was conducted in accordance with the Declaration of Helsinki, and the protocol was approved by the Ethics Committee of Institutional Review Board of KCDC (2013-07CON-03-4C, 2013-12EXP-03-5C). Since 2015, the KNHANES has been exempted from review following the Bioethics and Safety Act.

### 2.2. Variables

Household income level was determined as the gross household income, divided by the square root of the number of household members and categorized as low, low-middle, middle-high, or high for family size. Educational level was categorized as junior high school, high school, or college and more.

Among the lifestyle variables, smoking status was classified as current smoker, past smoker, and non-smoker. Sleep duration was classified into three groups based on an average sleep time of 6.8 h in this study: inadequate sleep (less than 5 h); normal sleep duration (6 to 7 h); excessive sleep (more than 8 h).

The community periodontal index (CPI) was used to measure periodontitis in the KNHANES, and the oral cavity was divided into the following sextants: #18–14, #13–23, #24–28, #34–38, #33–43, and #44–48. CPI was selected as a tool for evaluating the periodontal health status of population by the World Health Organization (WHO) [20]. The CPI was rated on the following scale from 0 to 4: 0 = normal, 1 = gingivitis with bleeding on probing, 2 = presence of calculus, 3 = probing depth (PD) of 4–5 mm, 4 = PD of 6 mm or more. CPI grades 1 to 2 were defined as absence of periodontitis and CPI grades 3 to 4 were defined as periodontitis. If CPI grades 3 or 4 were present in at least one of the sextants examined for periodontal health, the participant was regarded as a patient with periodontitis. Periodontal assessment was conducted by calibration-trained dentists using a CPI probe (WHO CPI probe, Osung, Korea). Measurements of CAL, pocket depth, and periodontal treatment status were not performed in the KNHANES (2013–2015).

Dependent variables were classified as non-diabetic (fasting serum glucose: FSG < 100 mg/dL), fasting serum glucose disorder (100 ≤ FSG ≤ 125 mg/dL), and diabetic (FSG ≥ 126 mg/dL or taking hypoglycemic agent or insulin injection). In this study fasting serum glucose disorder means only impaired glucose not diagnosed with diabetes, and it means prediabetes.

### 2.3. Statistical Analysis

Statistical analyses were performed with the Statistical Package for the Social Sciences (SPSS), version 25 (IBM SPSS Statistics for Windows, Armonk, NY, USA). A plan file was produced by calculating stratified variables with a layer for dispersion estimation, clustered enumeration districts, and weighted samples with an integral weight of 3 years of existing examination survey–nutrition relation weight. Participants were divided into three groups on the basis of the presence or absence of DM and prediabetes. Each group was then divided into a non-periodontitis group and a periodontitis group, according to the CPI value. The chi-squared test was used for assessing the periodontitis status of participants and demographic socioeconomic variables. Statistically significant variables were analyzed by complex-sample multinomial logistic regression analysis to evaluate the odds ratio (OR) and 95% confidence interval (CI). Complex-samples multinomial logistic regression analysis was performed to assess the association between sleep duration and prediabetes and diabetes in participants with periodontal disease. Sex, age, education level, household income level, smoking status, and average sleep duration were used as confounding variables.

## 3. Results

Of the participants, 25.7% had periodontitis. The prevalence of periodontitis by sex was 58.9% in male and 41.1% in female. The proportion of participants with periodontitis increased with age. Higher education and income levels were associated with a low prevalence of periodontitis. Average sleep duration was 6.8 h in Korean adults. The prevalence of periodontitis for those with ≤5 and ≥8 h of sleep was 16.2% and 17.8%, respectively. In assessments based on smoking status, 23.5% of participants with periodontitis were past smokers and 30.9% were current smokers. Among the participants with periodontitis, 28.6% had prediabetes and 14.2% had diabetes. Among those without periodontal disease, 17.4% had prediabetes and 4.5% had diabetes (Table 1).

Among participants with periodontitis, in comparison with those with a normal sleep duration of 6–7 h, participants with an average sleep duration of less than 5 h showed a 1.49-fold higher prevalence of diabetes while those with an average sleep duration of ≥8 h showed a 1.57-fold higher prevalence of diabetes. Among participants without periodontitis, in comparison with participants with normal sleep duration, those with an average sleep duration of <5 h showed 1.25-fold and 1.53-fold higher prevalence of prediabetes and diabetes, respectively (Table 2 and Table 3). The confounding variables were not controlled in Table 2 and Table 3.

To investigate the effect of sleep duration on the prevalence of diabetic participants with periodontitis, a complex-samples multinomial logistic regression analysis was performed after adjusting the findings for sex, age, socioeconomic variables, and smoking (Table 4). Periodontitis increased the prevalence of prediabetes and DM in total. The prevalence of prediabetes and DM adjusted for sleep duration was 1.28-fold higher and 1.86-fold higher in participants with periodontitis. The prevalence of diabetes was 1.49-fold higher in periodontitis participants with an average sleep duration of >8 h. However, in participants without periodontitis with a sleep duration of ≥8 h, the prevalence of diabetes was 1.57-fold higher than that in participants with a normal sleep duration of 6–7 h. Moreover, the diabetes incidence was 1.49-fold higher in participants without periodontitis with sleep duration of ≤5 h compared to that in participants with normal sleep duration of 6–7 h.

## 4. Discussion

The pathophysiologic mechanism underlying the association between sleep and DM is likely to be complex and bidirectional. Epidemiological evidence on this link, however, is not yet conclusively established. Therefore, we investigated the effects of sleep duration on impaired fasting glucose levels and diabetes in participants with periodontal disease by using data from the Sixth KNHANES (2013–2015). We found that altered sleep duration could be a risk factor for an increased incidence of diabetes regardless of periodontitis, and that proper sleep duration is important for diabetes.

The sleep–wake cycle is a complex phenomenon that encompasses several physiological and behavioral oscillations [21]. Sleep is a biological process that is essential for all human beings and is the most basic requirement for a healthy daily life. Insufficient sleep duration increases the risk of premature death [22]. In the normal sleep–wake cycle, immune system indicators like the number of undifferentiated naïve T cells and the production of pro-inflammatory cytokines exhibit peaks during early nocturnal sleep, whereas circulating numbers of immune cells with immediate effects or functions, like cytotoxic natural killer cells, as well as anti-inflammatory cytokine activity peak during daytime wakefulness [4]. Thus, sleep health may be promoted for therapeutic control of chronic infections and inflammatory and neuropsychiatric diseases [23].

In addition, short sleep duration could be a significant risk factor for diabetes [6]. Sleep deprivation has become a global phenomenon, and short sleep duration adversely affects human physical health [24]. Adequate amount and quality of sleep are important for metabolic control in patients with type 2 diabetes. Too short or too long sleep durations disrupt glycemic control in both prediabetic and type 2 diabetic patients. The development of impaired glucose tolerance is associated with periodontitis. Periodontitis is more common in diabetes patients and worsens with this condition. In particular, duration of diabetes, fasting serum glucose, glycated hemoglobin (HbA1c) levels, and compliance to self-management of diabetes were significantly correlated with periodontal health among individuals with type 2 diabetes [25].

Periodontal disease is a chronic inflammatory disease characterized by the destruction of the tooth-supporting connective tissues in response to subgingival infection with various periodontal pathogens [14]. The risk of periodontitis has been reported to be 1.34 times higher in subjects with a sleep duration of less than 7 h per day on average [26]. In this study, however, the prevalence of diabetes associated with periodontal disease was observed in participants with sleep durations <5 h or with more >8 h than in participants with normal sleep duration. The prevalence of diabetes was 1.49-fold higher in periodontitis participants with an average sleep duration of <5 h compared to that in participants with a sleep duration of 6–7 h, while the prevalence was 1.57-fold higher in those with a typical average sleep duration of ≥8 h. In the case of fasting serum glucose, the prediabetes stage, the incidence of prediabetes without periodontal disease was higher with longer sleep duration than in participants with proper sleep duration. Sleep problems were associated with disturbances in cortisol responses to stress, as well as changes diurnal cortisol output in people with DM [27]. The increasing level of cortisol make it difficult to control fasting serum glucose in DM. Therefore, sleep quality deteriorated due to more than usual or insufficient sleep can lead to an increase in prediabetes and DM. The results of this study were similar to previous result of another study outlining the relationship between sleep duration and periodontitis in Korean adult women [28].

Nakada et al. reported that fatigue worsened systemic health in rats and increased gingival inflammation and alveolar bone loss in experimental periodontitis [29]. In our study, the prevalence of diabetes was 1.49-fold higher in periodontitis participants with an average sleep duration of ≥8 h than that in participants with normal sleep duration. However, corresponding prevalence in participants without periodontitis who slept for ≥8 h was 1.57-fold higher compared to that in participants with a normal sleep duration. Moreover, the diabetes incidence was 1.49-fold higher in participants without periodontitis and sleep duration ≤5 h compared to that in participants with normal sleep duration. Dumitrescu et al. reported that the disturbed sleep index was associated with oral health status and behavior [30]. This study had some limitations as it was a cross-sectional analysis and based solely on information gained from KNHANES-VI. Although we analyzed the prevalence of prediabetes and DM in participants without periodontitis and with periodontitis, more various variables should be considered to establish the accurate relationship as type 2 diabetes is a multifactorial disease. Furthermore, it is necessary to study variables that may be associated with hormones as well as prediabetes and DM.

## 5. Conclusions

Our findings suggested that periodontitis can increase the risk of prediabetes and DM. We also investigated the effects of sleep duration on prediabetes and DM in participants with periodontitis and non-periodontitis. We found that longer/shorter sleep duration may be a risk factor for diabetes and that the proper sleep duration could be good for prevention of diabetes. It is necessary to develop a comprehensive health promotion program that can improve the sleeping habits of adults and to combine oral health programs to prevent periodontitis and diabetes.

## Figures and Tables

**Table 1:** The prevalence of periodontitis by demographic and socioeconomic parameters, smoking status, and sleeping duration.

	Total (%)			No Periodontitis (%)			Periodontitis (%)			*p*-Value
Variable	DM	IFG	NDM	DM	IFG	NDM	DM	IFG	NDM	
Sex										<0.001
Male	8.4	24.4	67.2	5.3	21.5	73.3	15.6	31.2	53.3	
Female	5.7	16.2	78.2	3.9	13.9	82.2	12.3	24.9	62.9	
Age group (years)										<0.001
19–34	1.1	9.1	89.8	0.9	9.0	90.1	3.5	10.2	86.3	
35–44	4.5	20.7	74.8	3.6	18.6	77.7	7.3	28.1	64.5	
45–54	8.3	26.8	64.8	5.8	24.4	69.8	12.6	31.0	56.4	
55–64	16.0	31.8	52.2	12.0	30.8	57.2	20.9	33.0	46.2	
>65	21.2	28.3	50.5	19.6	28.6	51.8	22.8	28.1	49.2	
Education level										<0.001
≤Junior high school	16.2	28.9	55.0	13.4	26.0	60.5	19.4	32.2	48.5	
High school	5.5	20.0	74.5	3.2	17.3	79.5	12.9	28.6	58.5	
≥College	4.6	16.8	78.6	3.2	15.0	81.8	10.4	24.7	64.9	
Household income level										<0.001
Low	13.3	22.2	64.5	7.7	19.2	73.1	22.8	27.2	50.1	
Low-Middle	8.4	19.6	72.0	5.6	17.3	77.1	15.9	25.6	58.4	
Middle-High	5.4	20.2	74.5	3.8	16.7	79.5	10.5	31.1	58.4	
High	5.6	20.3	74.1	3.8	17.7	78.6	12.1	29.3	58.6	
Smoking status										<0.001
Current smoker	7.7	24.1	68.2	4.2	21.0	74.8	14.3	29.8	55.9	
Past smoker	9.7	25.4	64.9	6.1	23.0	70.9	17.5	30.7	51.7	
Non-smoker	5.9	17.1	77.0	4.2	14.7	81.1	12.5	26.6	60.9	
Estimate sleeping duration										<0.001
≤5 h	9.3	23.2	67.5	6.3	20.3	73.4	16.2	30.0	53.8	
6–7 h	6.2	20.3	73.5	4.4	17.3	78.3	11.9	29.4	58.7	
≥8 h	7.5	18.8	73.6	4.1	16.4	79.5	17.8	26.0	56.1	

DM: diabetes mellitus; IFG: impaired fasting glucose; NDM: no diabetes mellitus.

**Table 2:** The prevalence of fasting serum glucose disorder and diabetes without periodontitis.

Variables	IFG	DM
	Crude OR (95% CI)	Crude OR (95% CI)
Sex		
Male	**1.74 (1.53–1.99)**	**1.51 (1.21–1.90)**
Female	1.00	1.00
Age	**0.61 (0.58–0.65)**	**0.41 (0.38–0.45)**
Education level		
≤Junior high school	**2.35 (1.97–2.81)**	**5.66 (4.27–7.51)**
High school	**1.19 (1.03–1.39)**	1.03 (0.77–1.37)
≥College	1.00	1.00
Household income level		
Low	1.17 (0.92–1.47)	**2.20 (1.55–3.11)**
Low-Middle	1.00 (0.83–1.19)	**1.51 (1.12–2.03)**
Middle-High	0.93 (0.79–1.10)	0.99 (0.73–1.36)
High	1.00	1.00
Smoking status		
Current smoker	**1.55 (1.30–1.84)**	1.07 (0.78–1.47)
Past smoker	**1.79 (1.50–2.13)**	**1.66 (1.24–2.23)**
Non-smoker	1.00	1.00
Estimate sleeping duration		
≤5 h	**1.25 (1.03–1.52)**	**1.53 (1.14–2.07)**
≥8 h	0.94 (0.80–1.10)	0.91 (0.70–1.20)
6–7 h	1.00	1.00

IFG: impaired fasting glucose; DM: diabetes mellitus; independent variable: diabetes mellitus (ref. non-diabetic patient); cOR: crude odds ratio; 95% CI: 95% confidence interval; crude odds ratios and 95% confidence interval were estimated by complex-samples multinomial logistic regression analysis; Bold letters indicate statistically significant differences.

**Table 3:** The prevalence of fasting serum glucose disorder and diabetes with periodontitis.

Variables	IFG	DM
	Crude OR (95% CI)	Crude OR (95% CI)
Sex		
Male	**1.48 (1.22–1.79)**	**1.50 (1.18–1.90)**
Female	1.00	1.00
Age	**0.76 (0.70–0.82)**	**0.56 (0.51–0.63)**
Education level		
≤Junior high school	**1.74 (1.37–2.22)**	**2.48 (1.82–3.38)**
High school	**1.28 (1.01–1.64)**	1.37 (0.98–1.91)
≥College	1.00	1.00
Household income level		
Low	1.08 (0.81–1.45)	**2.21 (1.56–3.12)**
Low-Middle	0.88 (0.68–1.14)	1.32 (0.96–1.83)
Middle-High	1.06 (0.83–1.36)	0.87 (0.61–1.23)
High	1.00	1.00
Smoking status		
Current smoker	1.22 (0.97–1.53)	1.24 (0.93–1.67)
Past smoker	1.36 (1.07–1.72)	**1.65 (1.25–2.19)**
Non-smoker	1.00	1.00
Estimate sleeping duration		
≤5 h	1.11 (0.85–1.46)	**1.49 (1.09–2.04)**
≥8 h	0.93 (0.74–1.16)	**1.57 (1.19–2.07)**
6–7 h	1.00	1.00

IFG: impaired fasting glucose; DM: diabetes mellitus; independent variable: diabetes mellitus (ref. non-diabetic patient); crude OR: crude odds ratio; 95% CI: 95% confidence interval; crude odds ratios and 95% confidence interval were estimated by complex-samples multinomial logistic regression analysis; bold letters indicate statistically significant differences.

**Table 4:** Effect of sleep duration on fasting serum glucose and diabetes with periodontitis and non-periodontitis.

Variables			IFG	DM
			Adjusted OR (95% CI)	Adjusted OR (95% CI)
Total	Periodontitis	Yes	**1.28 (1.13–1.46)**	**1.86 (1.55–2.23)**
		No	1.00	1.00
	Sleep duration	≤5 h	1.09 (0.92–1.30)	1.17 (0.93–1.48)
		≥8 h	1.03 (0.90–1.18)	**1.29 (1.05–1.57)**
		6–7 h	1.00	1.00
Non-Periodontitis	Sleep duration	≤5 h	1.12 (0.91–1.38)	**1.49 (1.09** **–2.04)**
		≥8 h	1.13 (0.95–1.34)	**1.57 (1.19** **–2.07)**
		6–7 h	1.00	1.00
Periodontitis	Sleep duration	≤5 h	1.06 (0.80–1.41)	1.24 (0.89–1.73)
		≥8 h	0.90 (0.72–1.14)	**1.49 (1.12** **–1.98)**
		6–7 h	1.00	1.00

IFG: impaired fasting glucose; DM: diabetes mellitus; independent variable: diabetes mellitus (ref. non-diabetic participants); adjusted for sex, age, educational level, income level, smoking status; adjusted OR: adjusted odds ratio; 95% CI: 95% confidence interval. Adjusted odds ratios and 95% confidence intervals were estimated by complex-samples multinomial logistic regression analysis; Bold letters indicate statistically significant differences.
